# Enhancing the Stability of Fungal Lipases by Immobilization onto Accurel MP 1000 Support and Additional Glutaraldehyde Crosslinking

**DOI:** 10.3390/biom15101372

**Published:** 2025-09-26

**Authors:** Alexandra Kovács-Kotogán, Tamás Papp, Csaba Vágvölgyi, Miklós Takó

**Affiliations:** 1 Department of Biotechnology and Microbiology, Faculty of Science and Informatics, University of Szeged, Közép fasor 52, H-6726 Szeged, Hungary; primula15@gmail.com (A.K.-K.); pappt@bio.u-szeged.hu (T.P.); csaba@bio.u-szeged.hu (C.V.); 2 HUN-REN-SZTE Pathomechanisms of Fungal Infections Research Group, University of Szeged, Közép fasor 52, H-6726 Szeged, Hungary

**Keywords:** microbial lipases, adsorption, polypropylene hydrophobic support, glutaraldehyde, reusability, stability properties

## Abstract

Commercial fungal lipases from *Rhizopus oryzae*, *Rhizopus niveus*, *Aspergillus niger*, *Rhizomucor miehei*, and *Candida rugosa* were immobilized via physical adsorption onto Accurel MP 1000, a hydrophobic polypropylene support. The effects of enzyme concentration, pH, temperature, and glutaraldehyde post-treatment were systematically evaluated. Immobilization generally enhanced enzyme stability, which was further improved in several cases by glutaraldehyde crosslinking. The immobilized preparations retained over 50% of their initial activity for 3–6 cycles, and 7–10 cycles following glutaraldehyde treatment. While soluble enzymes lost nearly all activity within three months at 5 °C and 25 °C and retained only 5–20% at −20 °C, the immobilized forms preserved 50–100% of their activity under all storage conditions tested. Immobilized lipases also exhibited improved thermal stability at 60 °C by general increments between 1.3 and 1.8 times compared to soluble lipases. Increased tolerance to pH fluctuations was observed in most immobilized enzymes, particularly from *R. oryzae*, *R. niveus*, *R. miehei*, and *C. rugosa*. Organic solvent tolerance of the immobilized enzymes showed highest stability in hexane (66–100% residual activity after 4 h incubation). Glutaraldehyde treatment affected solvent stability of immobilized lipases in enzyme and solvent dependent manner. These findings demonstrate the improved stability and applicability of the produced biocatalysts in varying reaction environments.

## 1. Introduction

Enzymes and their use as biocatalysts have received great attention because enzyme-catalyzed reactions, compared to chemical catalysis, can occur under relatively mild conditions with high reaction rate and selectivity [1]. However, despite all these advantages, the industrial use of enzymes is often hindered by the difficulty of low thermal and chemical stability and the lack of recovery and reusability in multiple reactions. Immobilization of enzymes in an appropriate support can reduce the risk of these activity-limiting factors during reactions [2,3]. The immobilization could improve enzyme activity and stability, ensure their reusability in several reaction cycles, and facilitate the separation of the catalyst from the product. Therefore, industry has high demand for immobilized enzyme preparations to avoid limitations of applying soluble enzymes. Since the use of immobilized enzymes can reduce the cost of the biocatalytic process, they are more economically applicable in the industry than soluble forms [4].

Lipases (EC 3.1.1.3) are widely used biocatalysts in the industry due to their ability to catalyze various types of reactions, such as triacylglycerol hydrolysis, trans- and interesterification reactions, alcoholysis and aminolysis [5]. Lipases are applied in the detergent, food, pharmaceutical, cosmetic, pulp and paper and oleochemical industries, as well as in the biodiesel production [6]. Most soluble lipases are generally unstable in anhydrous media and can be quickly denatured under pH and temperature conditions; therefore, harsh industrial conditions can reduce their shelf-life [7]. Due to the high industrial interest, there is great demand for immobilized and stable lipase preparations. For instance, immobilized lipases were frequently used in the processing of edible oils, dairy products as well as various food additives in the food industry [8].

Among the techniques used today, an efficient method for immobilizing lipase enzymes is the adsorption to hydrophobic matrices [8,9]. Generally, physical adsorption of enzymes is a simple, economical and green technique, since binding does not require a special immobilization procedure or chemicals [4,10]. Accurel MP 1000 (<1500 µm particle size) is a hydrophobic polypropylene porous polymer by which a nonspecific hydrophobic interaction could be established. The average diameter of the pores of the matrix is 23 nm and has a large external and internal surface of approximately 39 m^2^/g due to its porosity [11]. Lipases with a typical diameter of 5 nm [12] can easily adsorb on the outer surface as well as inside the pores. The lipase recognizes and connects to the surface of the hydrophobic support and assumes an active conformation, as in the phenomenon of interfacial activation in the case of natural hydrophobic substrates. The hydrophobicity of polypropylene has a significant effect on enzyme activity after immobilization [13]. As a very hydrophobic material, the Accurel MP 1000 cannot adsorb polar compounds such as glycerol. This feature prevents the formation of a hydrophilic layer around the enzyme by glycerol, which can cause diffusion limitation during the migration of lipids to the enzyme’s active site in esterification and transesterification reactions [14]. Owing to these advantageous properties, Accurel MP 1000 has become a widely used support material in recent lipase immobilization studies [15,16,17,18,19,20,21,22]. A relatively weak and reversible binding between the enzyme and the support is the disadvantage of adsorption, resulting in a possible rapid leakage for the enzyme [2].

Glutaraldehyde is a commonly used protein crosslinking agent since it can establish an interaction between lysine residues of enzyme proteins, enabling immobilization without a support [4,23,24]. Furthermore, it can be combined with support-based immobilization techniques where enhanced immobilization efficiency and enzyme stability can be achieved in two different ways [25]: (a) the support can be preactivated by glutaraldehyde resulting in a glutaraldehyde-amino activated support that can facilitate a fast ionic-exchange [26] or (b) modification of the enzyme-support complex after immobilization establishing a crosslink between bound enzymes [27]. Adsorption and subsequent crosslinking with glutaraldehyde could avoid leaching of adsorbed enzyme. Although Accurel MP 1000–lipase complexes post-treated with glutaraldehyde have been previously employed for specific reactions [18,28,29], the impact of such treatment on enzymatic activity, stability, and immobilization efficiency has been comparatively less investigated for this support.

In our previous research, we successfully developed a lipase–Accurel MP 1000 complex that demonstrated high catalytic activity and stability in both hydrolytic and transesterification reactions [30]. Our subsequent study investigated the hydrolytic capacity of commercially available *Rhizopus oryzae*, *Rhizopus niveus*, *Aspergillus niger*, *Rhizomucor miehei*, and *Candida rugosa* lipases using various edible oils as substrates [31]. All enzymes demonstrated excellent catalytic efficiency on vegetable oils, and *R. miehei* lipase also facilitated the release of bioactive fatty acids, such as α-linolenic acid, eicosapentaenoic acid, and docosahexaenoic acid from menhaden fish oil, in liquid-phase reactions [31]. Building on these findings, the present study aimed to immobilize the same commercial lipases onto Accurel MP 1000 polypropylene support via an optimized physical adsorption protocol. The kinetics of enzyme binding under various conditions were evaluated, and the resulting biocatalysts were biochemically characterized. Furthermore, the impact of glutaraldehyde post-treatment on biocatalyst stability was assessed. The results indicated that immobilization induced notable changes in several enzymatic properties compared to the soluble forms.

## 2. Materials and Methods

### 2.1. Materials

Lipases from *R. oryzae* (product no. 62305), *R. niveus* (product no. 62310), *A. niger* (product no. 62301), *R. miehei* (formerly known as *Mucor miehei*; product no. 62298) and *C. rugosa* (product no. 62316) and the lipase substrate *p*-nitrophenyl palmitate (*p*NPP) were purchased from Sigma-Aldrich (Munich, Germany). Accurel MP 1000 support (particle size < 1500 μm) derived from 3M Deutschland GmbH (Neuss, Germany). Organic solvents, i.e., dimethyl sulfoxide (DMSO), methanol, ethanol, propanol, hexane and glutaraldehyde 25% (*w*/*w*) were acquired from VWR International (Debrecen, Hungary).

### 2.2. Lipase Activity Assay

The soluble lipase activity was determined by following the standard *p*NPP-based method used in our previous work [32]. The increase in absorbance at 405 nm was measured produced by the released *p*-nitrophenol in the hydrolysis of 0.75 mM *p*NPP in 50 mM sodium phosphate buffer (pH 7.0) at 30 °C in 30 min. The activity of *A. niger* lipase was determined in acetate buffer (50 mM; pH 4.5 and 5.5).

The lipolytic activity of the immobilized enzymes was measured similarly to that of the soluble enzyme, but with some modifications. First, an appropriate amount of biocatalysts (0.1–20 mg), 250 μL of buffered substrate (1.5 mM *p*NPP in 50 mM phosphate buffer, pH 7.0) and 250 μL of phosphate buffer (50 mM, pH 7.0) were mixed in 1 mL Eppendorf tubes. The mixtures were incubated at 30 °C for 1 h with frequent stirring, then 200 µL of the supernatant was transferred to the wells of a 96-well microtiter plate. The reaction was stopped with 50 μL of 0.1 M sodium carbonate. The liberated *p*-nitrophenol was determined by SPECTROstar Nano (BMG Labtech, Offenburg, Germany) microplate reader. One enzymatic unit (U) was defined as the amount of enzyme that catalyzes the formation of one μmol of *p*-nitrophenol in one minute under the conditions of the assay. Reactions were performed in at least three independent experiments.

### 2.3. Immobilization of the Lipases

Lipase immobilization was carried out as described in Kotogán et al. [30] set up by the method of Alnoch et al. [7]. Briefly, a mass of 200 mg of Accurel MP 1000 polypropylene matrix was stirred in 50% ethanol for 30 min, then the particles were filtrated on a Whatman filter paper (Sigma-Aldrich, Munich, Germany) and washed with distilled water. Dehydration of the support must be avoided; otherwise, low enzyme binding may occur. The activated support was mixed with an appropriate concentration of 10 mL (from 0.001 to 1 mg/mL) of enzyme solution in 25 mM phosphate buffer (pH 7.0) or 25 mM acetate buffer (pH 4.5) (for *A. niger* lipase). The mixture was incubated under gentle shaking (200 rpm) at 25 °C for 1 h to 24 h. After incubation, the biocatalyst was filtrated and washed several times with distilled water, then lyophilized and stored at −20 °C until use. To follow the loss of activity during the adsorption, a control enzyme solution (without support) was also prepared and incubated under the above-mentioned conditions. Adsorption was monitored by measuring the residual hydrolytic activity (and protein concentration, see Section 2.4) in the supernatant. The percentage immobilization efficiency and activity yield were calculated based on the following equations:

Immobilization efficiency (%) = [(A−B)/A] × 100

Activity yield (%) = (C/A) × 100

where A is the total *p*NPP-hydrolyzing activity (U) of the enzyme solution prior to immobilization, B is the total *p*NPP-hydrolyzing activity (U) remained in the solution after immobilization, and C is the total *p*NPP-hydrolyzing activity (U) detected on the 200 mg support. Immobilized activity shows the enzyme activity relative to one mg of Accurel MP 1000, expressed as U/mg support.

The effect of binding conditions (pH and temperature) on immobilization efficiency and immobilized activity was also tested at pH 5.5 acetate buffer (25 mM) and pH 8.5 Tris-HCl buffer (25 mM) as well as at 5 °C. The immobilized activity of the biocatalysts prepared under standard condition (pH 7.0 or 4.5 and 25 °C) was taken as 100%.

### 2.4. Protein Concentration Assay

Soluble enzyme protein was determined using a Qubit Fluorometer (Invitrogen, Waltham, MA, USA) and the Quant-iT Protein Assay Kit (Invitrogen, Waltham, MA, USA) according to the manufacturer’s instructions. Immobilization yield (%) was calculated from the ratio between the amount of immobilized protein (difference between protein content of initial enzyme solution and supernatant after immobilization) and initial protein content in enzyme solution.

### 2.5. Glutaraldehyde Treatment of the Biocatalysts

The filtrated and washed immobilized lipase preparations were incubated in a solution of 1, 2 or 3% (*v*/*v*) glutaraldehyde in 50 mM sodium phosphate buffer (pH 7.0) at 25 °C for 1 h, under continuous mild stirring. The biocatalysts were then filtrated and washed with distilled water to remove glutaraldehyde. After lyophilization, the complexes were stored at −20 °C. The activity of nontreated biocatalysts was taken 100%. Stirring in glutaraldehyde-free sodium phosphate buffer (pH 7.0) served as a control.

### 2.6. Characterization of Immobilized Lipases

#### 2.6.1. Reusability of the Complexes

The glutaraldehyde treated and untreated biocatalysts were tested successively 10 times in standard hydrolytic reactions based on *p*NPP (30 °C, pH 7.0, 1 h incubation). After each cycle, the complexes were filtrated and washed thoroughly with distilled water. Then, the surrounding reaction mixture was replaced with fresh medium. The activity of the freshly prepared biocatalyst was considered 100%.

#### 2.6.2. Storage Stability Test

The glutaraldehyde treated and untreated biocatalysts and soluble lipases were stored at −20 °C, 5 °C or 25 °C for three months, then residual activities were established. The activities of fresh soluble enzyme solutions and immobilized enzymes without storage were considered 100%.

#### 2.6.3. Thermal Stability

The glutaraldehyde treated and untreated biocatalysts and soluble lipases were incubated at 60 °C or 80 °C in 50 mM phosphate buffer (pH 7.0). After 0.5, 2, 6, 12 and 24 h of preincubation, standard activity determination was carried out as described above. The activity of the corresponding soluble and immobilized enzymes without preincubation was considered as 100%.

#### 2.6.4. pH Stability Test

The glutaraldehyde treated and untreated biocatalysts and soluble lipases were incubated in 50 mM buffers of acetate (pH 5.5), phosphate (pH 7.0) or Tris-HCl (pH 8.5) for 24 h at 5 °C. After preincubation, residual activities were determined according to the standard assay. The activity of the corresponding soluble and immobilized enzymes without preincubation in buffers was considered as 100%.

#### 2.6.5. Organic Solvent Stability Test

To test the solvent stability of the immobilized lipase preparations, complexes were incubated for 4 h or 24 h at 5 °C in concentrated organic solvents with different hydrophobicity values (logP: partition coefficient *n*-octanol/water) such as DMSO (logP = −1.3), methanol (−0.76), ethanol (−0.3), *n*-propanol (0.25) and *n*-hexane (3.5). LogP values originate from the data of solvent manufacturer and Sangster [33] and Kotogán et al. [34]. After preincubation, the solvents were removed by pipetting, and the biocatalysts were dried under a laminar flow box. Residual activities were evaluated using the standard activity determination method (see Section 2.2). The activity of the biocatalysts without preincubation in solvents was taken as 100%.

### 2.7. Statistical Analysis

Assays were performed in three independent experiments and values were expressed as an average of the replicates together with percent coefficient of variation or standard deviation data. Statistical analysis of the results was performed by multiple *t*-test with false discovery rate (FDR) (Q = 10%), or one-way ANOVA followed by Tukey’s multiple comparison test using the GraphPad Prism 8.00 software (GraphPad Software Inc., San Diego, CA, USA). A *p* value less than 0.05 was considered as statistically significant. Pearson’s correlation coefficients (Pearson r) were calculated using Microsoft Excel 365 function.

## 3. Results and Discussion

### 3.1. Immobilization of Lipases to Accurel MP 1000

#### 3.1.1. Optimization of the Binding

The soluble lipase preparations used for the study showed different hydrolytic activity against the *p*NPP substrate. Namely, at 1 mg/mL concentration, the lipases of *A. niger*, *R. niveus*, *R. oryzae*, *R. miehei*, and *C. rugosa* demonstrated volumetric activities of 7.15, 212.2, 1697, 2231 and 2703 U/mL, respectively, under the standard enzyme activity assay (pH 7.0, 30 °C). As seen, the activity of *A. niger* lipase was much lower than that of the other enzymes tested, which may be due to the fact that the pH optimum of *Aspergillus* lipases is mostly in the acidic range [35]. Consistent with this, *A. niger* lipase was also tested at a lower pH, i.e., at pH 5.5 and 4.5, resulting in 113.6 and 238.5 U/mL activities, respectively. Therefore, the acetate buffer condition of pH 4.5 was considered standard for *A. niger* lipase in future reactions.

Physical and chemical state, and molecular size of the enzyme can also affect the immobilization [24]. With this context, molecular weights of ~45, ~83, ~43, ~67, and ~31 kDa were documented for the *A. niger*, *R. niveus*, *R. oryzae*, *C. rugosa*, and *R. miehei* lipases by the manufacturer (Sigma-Aldrich) and Urrutia et al. [36]. The spherical molecular diameter of *R. miehei* lipase was reported at around 3.5 nm [37], which corresponds to that value (~50 Å, 5 nm) typical of lipases [12]. Although the diameter of lipase molecules can double in an aqueous environment, their average diameter still ensures that lipase molecules can penetrate the pores and cover the available surface area of the Accurel MP 1000 support.

Many synthetic materials and polymers have been reported as effective support for lipase immobilization, as highlighted in the recent study by Guajardo [38]. Among these, Accurel MP 1000 is notable for its scalability and porous structure. Practical applications of lipases immobilized on widely used supports are summarized in the study of Mokhtar et al. [39]. Accurel MP 1000–lipase complexes, for instance, were used for the production of pharmacological derivatives [17], emollient esters [19], and structured lipids [20].

The immobilization started with an ethanolic solution (50%, *v*/*v*) pretreatment of the polypropylene support. Ethanol can enhance the effectiveness of immobilization, since it facilitates the penetration of lipases within the pores of Accurel, and removes the air from the support particles, which is favorable for adsorption [14,28,39]. Because the soluble enzyme preparations used have different hydrolytic activities, they were loaded onto a constant amount of support (200 mg), but at different concentrations ranging from 0.001 to 1 mg/mL for *R. oryzae*, *R. miehei*, and *C. rugosa*, and from 0.01 to 1 mg/mL for *A. niger* and *R. niveus* lipases (Table 1). The protein content of the commercial preparations was very low, that is, 1 mg/mL enzyme solutions contained only 21.8 to 143 µg/mL protein (Table 1). According to Gitlesen et al. [40], the commercial lipase preparations generally consist of about 10% of protein, i.e., a small fraction of the total mass is protein and only a part of this protein fraction is lipase. Kilcawley et al. [41] examined several commercially available peptidase and lipase preparations and found a low protein content for these preparations with respect to lipases between 0.5% and 11.1% on a dry weight basis. Commercial lipase and other enzyme preparations may contain many other materials which presumably play a role in the production process or in the stabilization of enzymes [41]. Sabbani et al. [12] and de Menezes et al. [19] also reported that the commercially available *C. rugosa* lipase preparation contains other proteins, polypeptides and polysaccharides beside the lipase protein, which could also influence the selectivity and catalytic activity of the enzyme. Although targeted enzyme protein isolation has not been performed in our current study, chromatographic purification of enzyme proteins can prevent these components from affecting immobilization and enzyme activity.

In our study, the protein content of the supernatant was also monitored during immobilization. Although different enzyme powder loads were tested (see Table 1), changes in protein concentration were studied for the highest enzyme concentration applied (1 mg/mL) (Table 2). For all enzymes studied, a decreasing trend in protein concentration of supernatants can be registered during immobilization process (Table 2). After incubation for 24 h, immobilization yields of 83%, 66%, 47% and 72% were achieved for *R. niveus*, *A. niger*, *R. miehei* and *C. rugosa* lipases, respectively. In *R. oryzae*, an immobilization yield of 79% was obtained after only 2 h of incubation. For comparison, loading of *C. rugosa*, *C. parapsilosis* and *R. oryzae* lipases onto Accurel MP 1000 support resulted in 100% [19], 80% [18,28] and 34.71% [29] immobilization yields, respectively, after about 18–24 h incubation. In other studies, protein adsorption rates of 36.76%, 35.4%, and 5.1% were observed for commercial porcine pancreatic lipase, and *Rhizopus arrhizus* and *R. miehei* fungal lipases, respectively, while binding to Accurel MP 1000 [42,43]. However, due to the low protein concentration in the lipase solutions (see Table 1), examination of the adsorption was confined to monitor the residual activity of the supernatant during immobilization.

**Figure 1:**
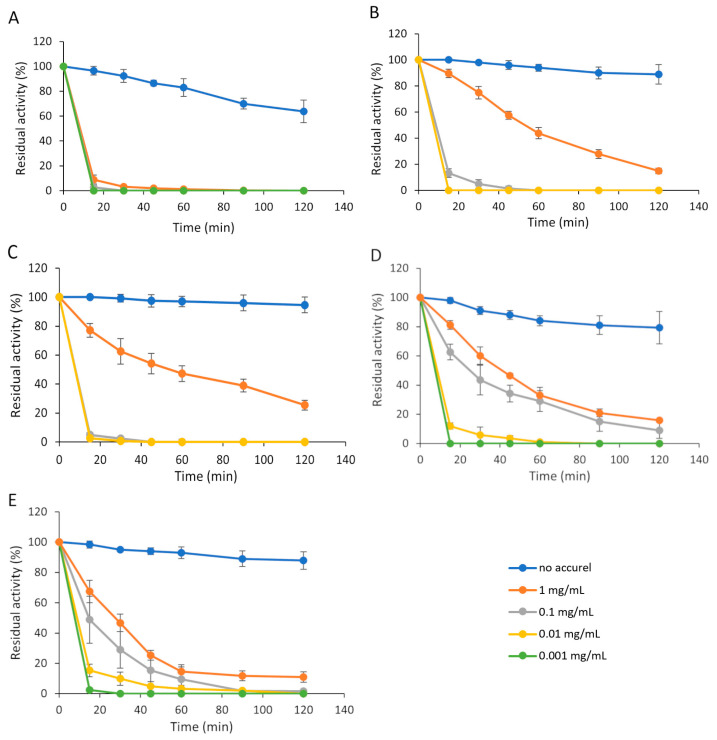
Residual activity of *R. oryzae* (**A**), *R. niveus* (**B**), *A. niger* (**C**), *R. miehei* (**D**) and *C. rugosa* (**E**) lipases in reaction surroundings during adsorption to Accurel MP 1000. Enzyme solution concentrations from 0.001 mg/mL to 1 mg/mL were used for immobilization. Blue line: incubation without solid support (1 mg/mL enzyme solution); red line: 1 mg/mL; grey line: 0.1 mg/mL; yellow line: 0.01 mg/mL; green line: 0.001 mg/mL enzyme solution. *R. oryzae*, *R. miehei* and *C. rugosa* lipases were loaded in concentration from 1 to 0.001 mg/mL, *R. niveus* and *A. niger* from 1 to 0.01 mg/mL. Results are presented as averages from three replicates; error bars represent standard deviation.

By varying the initial enzyme concentration, significant differences were found in both the rate and efficiency of immobilization (Table 1 and Figure 1). In general, at lower enzyme powder concentrations, the binding rate was very high in 15–30 min, while it was much lower at higher concentrations (0.1–1 mg/mL). In *R. oryzae* lipase, no residual activity was detected in the supernatant after 90 min of incubation at all enzyme concentrations applied due to the very rapid adsorption that occurred (Figure 1A). The immobilization of the other enzymes tested was slower, especially at concentration of 1 mg/mL where intensive adsorption can be observed in the earlier stage of incubation, then the intensity decreased significantly in the later period. For example, immobilization efficiency of 85% (15% residual activity) and 75% (25% residual activity) can be observed after 120 min incubation for the *R. niveus* and *A. niger* lipases, respectively, at 1 mg/mL powder concentration (Figure 1B,C), while the complete immobilization of these enzymes required of about 360 min incubation (Table 1). In *R. miehei* and *C. rugosa* lipases, more than 80% of immobilization efficiency was detected after 120 min of incubation at the highest enzyme powder concentration (Figure 1D,E), but considerable binding was not detected during the subsequent incubation period. After 24 h of incubation, immobilization efficiency of 93% and 97% were reached for *R. miehei* and *C. rugosa* lipases, respectively, when the initial powder concentration of the lipase solution was 1 mg/mL (Table 1).

A higher concentration of the initial powder solution resulted in a higher immobilized activity (Table 1). However, as the initial concentration of enzyme powder increased, the activity yield decreased substantially. In *Rhizopus* lipases, for example, high activity yield (121.2% and 115.2%) was detected at the lowest initial enzyme concentrations, while low activity yield (2.39% and 3.87%) was identified at 1 mg/mL loading powder concentration. For *A. niger*, *R. miehei*, and *C. rugosa* lipases, activity yield decreased to a lesser extent by 15.33–65.81%, by increasing the initial enzyme concentrations from 0.001 or 0.01 to 1 mg/mL. Relatively high immobilized activities of 25.07 and 21.18 U/mg support were found for *R. miehei* and *C. rugosa* lipases when the concentration of 1 mg/mL of enzyme solutions was used.

A possible reason for the decrease in the adsorption rate and low activity yield at high initial enzyme concentrations can be the saturation of the support during immobilization. As the number of available binding sites decreases, the initially rapid binding rate slows, leading to slower subsequent adsorption. This may result in the formation of a second enzyme layer on the support. Consequently, despite the potentially high amount of active immobilized enzyme, only moderate activity can be observed for the enzyme-support complex [7]. In addition, mass transfer (diffusion) limitations may arise due to high enzyme loading on the support, which can also contribute to reduced activity, as reported by Salis et al. [44]. The additional components, i.e., protein and sugar, which are present in commercial enzyme preparations, can also decrease the activity yield at higher concentration through adsorption to hydrophobic support and competitively occupy the binding sites.

To test the activity loss of soluble lipases during immobilization conditions, they were incubated at standard reaction conditions (see Section 2.3) used for immobilized enzyme preparation. Soluble lipases of *A. niger*, *C. rugosa*, and *R. niveus* were relatively stable, retaining more than 70% of their initial activity, while enzymes from *R. oryzae* and *R. miehei* retained 37.33% and 59.36% of their initial activity, respectively, after 4 h incubation under standard immobilization condition. The instability of the soluble *R. oryzae* enzyme may explain both the rapid decline in residual activity in the supernatant (Figure 1A) and the low bound activity observed for the immobilized enzyme. At low concentrations, each enzyme adsorbed rapidly (Figure 1A–E); therefore, the loss of activity was not significant in terms of activity yield due to short incubation period. However, this factor may contribute to the reduced activity yield observed at higher initial enzyme concentrations, where longer incubation is typically required.

Interestingly, in contrast to the soluble enzyme, which exhibited optimal activity in acidic conditions, the immobilized *A. niger* lipase showed approximately 30–40% lower activity at pH 4.5 compared to pH 7.0. A shift in the pH optimum toward a slightly alkaline region following immobilization has been reported in several studies and is commonly attributed to changes in the electrostatic environment of the enzyme upon binding to the support [45,46]. Consequently, in subsequent experiments, the activities of the immobilized enzyme preparations were consistently measured at pH 7.0 as well.

#### 3.1.2. Effect of pH and Temperature on Adsorption

It is well established that the physical adsorption of lipases onto hydrophobic supports is highly influenced by the ionizing properties of the buffer system; specifically, low ionic strength tends to favor lipase binding [47]. In this study, we focused on the effects of pH and temperature on the rate and efficiency of enzyme adsorption. Standard immobilization conditions were set at pH 7.0 and 25 °C for all enzymes, except for *A. niger* lipase, which was tested at pH 4.5. Initial enzyme concentrations were 0.1 mg/mL for *Rhizopus* and *A. niger* lipases, and 0.01 mg/mL for *R. miehei* and *C. rugosa* lipases.

The adsorption rates of *R. oryzae*, *R. niveus* and *C. rugosa* lipases at pH 8.5 were comparable to those at the standard pH (7.0), while adsorption at pH 5.5 proceeded more slowly (Table 3). Despite similar binding rates, immobilized activity for these enzymes was markedly lower when immobilization occurred under either acidic or alkaline conditions. Notably, immobilization at pH 8.5 led to a substantial decrease (37.3–77.3%) in bound activity. Similarly, *R. miehei* lipase displayed reduced bound activity (62.51%) at pH 8.5, while a notable improvement (about 44% increase) in activity was observed when immobilization was conducted at pH 5.5 (Table 3). A strong preference for acidic conditions was also showed for the *A. niger* lipase. When immobilized at pH 7.0, the resulting biocatalyst retained only 12% of the relative activity observed at pH 4.5.

It was reported that the pH during immobilization may affect both the reactivity of the enzyme toward the support and the orientation of the enzyme on the support after the adsorption [25]. While hydrophobic interactions are important driving forces in the adsorption of lipase onto supports such as EP-100 (the former name of Accurel MP 1000) [40], ionic interactions also play a significant role, making the pH of the working buffer used for adsorption another critical parameter. It was also reported that optimal adsorption often occurs near the isoelectric point (pI) of the enzyme [13].

Regarding temperature, our results showed that it primarily influenced the rate of adsorption rather than the final bound activity. At 5 °C, complete enzyme binding required 15–30 min longer than at room temperature; however, in most cases, the final immobilized activity was similar or only slightly reduced. An exception was observed with *R. miehei* lipase, which exhibited nearly double the bound activity when immobilized at 5 °C compared to 25 °C, under pH 7.0 condition (see Table 3). This enhanced activity may be due to slower enzyme inactivation at lower temperatures. While reduced temperature generally slows the adsorption kinetics, the observed increase in bound activity for *R. miehei* lipase suggests an enzyme-specific stabilization effect during low-temperature immobilization.

#### 3.1.3. Glutaraldehyde Treatment of Immobilized Enzymes

Immobilized lipases were treated with varying concentrations (1%, 2%, or 3%) of glutaraldehyde to crosslink the adsorbed lipase molecules on the support surface. The aim of this experiment was to evaluate the effect of glutaraldehyde chemical modification on the stability and activity of the biocatalysts. Following glutaraldehyde treatment, the enzymatic activities of the biocatalysts were measured. As controls, parallel immobilized samples were incubated under identical conditions in buffer without glutaraldehyde.

As shown in Table 4, a slight reduction in enzymatic activity was observed for all enzymes regardless of the glutaraldehyde concentration when compared to the initial biocatalysts (considered as 100% activity). The greatest activity loss was observed in the *A. niger* lipase preparation resulting in about 60% residual activity (*p* < 0.05), while *R. niveus* lipase exhibited the least sensitivity towards the incubation, retaining about 90% of its original activity.

Our results indicate that the observed loss of activity may not be attributed solely to the presence of glutaraldehyde. In *A. niger* and *R. miehei* lipases, for instance, control experiments revealed that significant (*p* < 0.05) activity loss also occurred after 1 h of incubation in glutaraldehyde-free phosphate buffer (pH 7.0, 25 °C) compared to initial biocatalysts (Table 4), suggesting that part of the activity decrease may be due to shear stress or destabilization caused by prolonged stirring in aqueous medium. For *R. oryzae*/*R. niveus*/*C. rugosa* lipase–Accurel complexes, this decrease in lipolytic activity was not significant (*p* > 0.05) (Table 4). Additionally, in all enzymes tested, there was no significant reduction (*p* > 0.05) in residual activities after glutaraldehyde treatment as compared to control incubation, implying that glutaraldehyde may partially stabilize the enzyme conformation and offset activity losses caused by incubation conditions. Although we have not yet investigated the presence of such bonds in the biocatalysts produced, this stabilizing effect is likely due to the formation of covalent bonds between the free amino groups of lysine residues, either within the enzyme or between adjacent enzyme molecules, via Schiff base formation [48]. Such crosslinking can reduce enzyme leaching and enhance structural rigidity, thereby contributing to improved retention of activity under operational conditions. This observation is consistent with findings by Zaak et al. [49], who reported residual activities of 63–97% for lipase and phospholipase octyl-Sepharose preparations following 1 h treatment with 0.1% (*v*/*v*) glutaraldehyde. Glutaraldehyde treatment of octyl-agarose beads also resulted in stable lipase biocatalysts in the study of Abellanas-Perez et al. [50], but the effect was strongly depended on the enzyme load applied for the immobilization. In cases of lowly loaded biocatalysts (1 mg/g), a slight increase in enzyme activity was observed following glutaraldehyde treatment [50]. For comparison, enzyme loadings between 0.5 mg/g (0.01 mg/mL) and 5 mg/g (0.1 mg/mL) were used in our corresponding studies depending on the tested enzyme (see Table 4 footnote). Glutaraldehyde has been shown to impart significant structural stability to other immobilized enzymes such as β-galactosidases by forming covalent bridges, resulting in enhanced operational and thermal stability [51,52]. Potential industrial applications of lipase–Accurel MP 1000 biocatalysts subjected to crosslinking with glutaraldehyde were documented for the production of milk fat substitutes [18], biodiesel [28] and ethyl lactate [29].

Based on the retained activity profiles, a glutaraldehyde concentration of 1% and 2% was selected for further treatments of *R. miehei*, and *R. niveus* and *C. rugosa* lipases, respectively, while 3% was found to be most suitable for *R. oryzae* and *A. niger* preparations.

### 3.2. Characterization of Biocatalysts

#### 3.2.1. Reusability

The reusability of the immobilized lipase preparations was evaluated to assess potential activity loss due to enzyme inactivation or leaching from the support during repeated reaction cycles. To this end, each preparation was subjected to 10 consecutive hydrolytic reactions using *p*NPP as substrate. The degree of hydrolysis was determined after each cycle, and residual activity was monitored.

**Figure 2:**
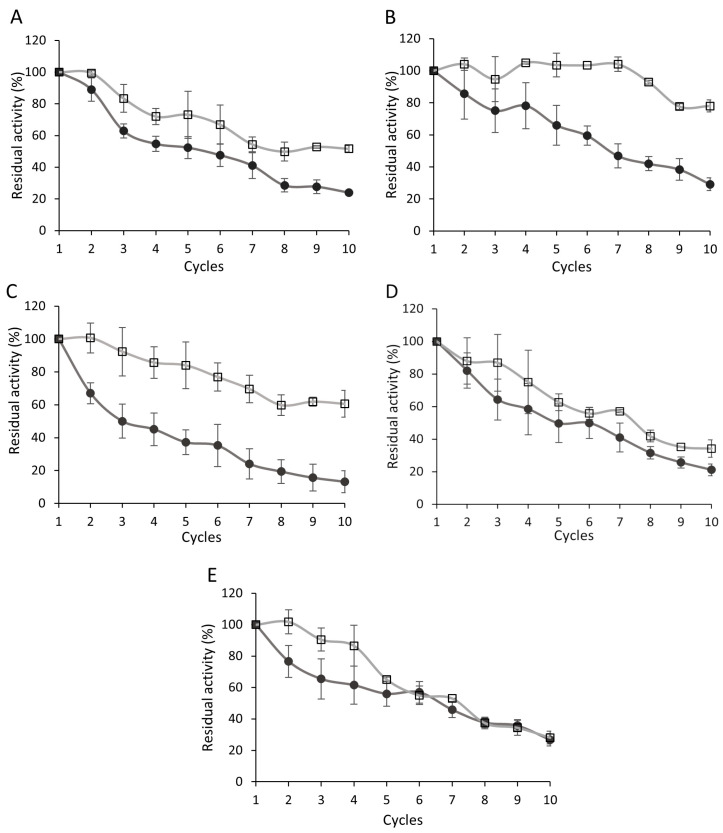
Reusability of the *R. oryzae* (**A**), *R. niveus* (**B**), *A. niger* (**C**), *R. miehei* (**D**) and *C. rugosa* (**E**) lipase–Accurel biocatalysts in 10 consecutive *p*NPP-based standard activity assays. ●: glutaraldehyde untreated biocatalyst; □: glutaraldehyde treated biocatalyst (*R. miehei* 1%, *R. niveus* and *C. rugosa* 2%, and *R. oryzae* and *A. niger* 3% (*v*/*v*) glutaraldehyde). Results are presented as averages from three replicates; error bars represent standard deviation.

Biocatalysts without glutaraldehyde treatment retained more than 50% of their initial activity through three to six reuse cycles (Figure 2A–E). Among these, *R. niveus* lipase demonstrated the highest operational stability, maintaining 29.2% of its initial activity after the 10th cycle (Figure 2B), while *A. niger* lipase exhibited the lowest, with only 13.2% residual activity remaining by the end of the test (Figure 2C).

In contrast, glutaraldehyde-treated biocatalysts showed markedly improved reusability. Residual activities were 10–50% higher than those of their untreated counterparts. Specifically, *R. oryzae* and *R. miehei* lipase preparations showed improvements of 10–25% (Figure 2A,D), while *R. niveus* and *A. niger* exhibited increases of 20–50% in residual activity (Figure 2B,C). The immobilized lipases from *R. oryzae*, *R. miehei*, and *C. rugosa* retained more than 50% of their original activity up to the seventh cycle, while *R. niveus* and *A. niger* maintained over 50% activity even after 10 cycles. For *C. rugosa*, no remarkable difference in residual activity between treated and untreated biocatalysts was observed after the fifth cycle, indicating a limited benefit of glutaraldehyde treatment in this case (Figure 2E). In a recent study, a *C. rugosa* lipase immobilized on mesoporous silica nanoparticles was also highly stable, retaining 63% of its initial activity after 10 cycles [53]. Comparable reusability results have been reported for *R. oryzae* lipase immobilized on NKA-9 resin and subsequently crosslinked with glutaraldehyde, where the enzymatic activity in acidolysis reactions declined below 50% after the seventh cycle [27].

Overall, the biocatalysts demonstrated satisfactory operational stability, and glutaraldehyde crosslinking further enhanced this property. This improvement is likely due to the formation of intermolecular covalent bonds between adjacent enzyme molecules on the support surface, which effectively reduces enzyme desorption and increases conformational rigidity [49]. Consequently, more robust and reusable immobilized enzyme systems can be achieved through post-immobilization glutaraldehyde treatment.

#### 3.2.2. Storage Stability

The long-term storage stability of the immobilized enzyme preparations was evaluated after three months of storage at −20 °C, 5 °C and 25 °C (Table 5). The soluble enzymes exhibited complete loss of activity at 5 °C and 25 °C and retained only 5–20% of their initial activity after storage at −20 °C. In contrast, the dry immobilized enzyme preparations displayed significantly better stability, showing activity losses of only 0–45% under all three temperature conditions.

Glutaraldehyde treatment of *R. miehei* and *C. rugosa* immobilized lipases did not result in a statistically significant improvement in storage stability compared to the untreated samples, regardless of storage temperature (Table 5). Similarly, no marked differences were observed between the glutaraldehyde treated and untreated *R. niveus* immobilized enzymes at either 5 °C or 25 °C. However, for the *A. niger* immobilized lipase, glutaraldehyde modification resulted in significantly lower residual activity at all storage temperatures when compared to the untreated preparation (*p* < 0.05). A slight reduction in stability was also detected for the *R. niveus* immobilized lipase following storage at −20 °C (*p* < 0.05). Conversely, glutaraldehyde treatment enhanced both the stability and residual activity of the *R. oryzae* immobilized lipase when stored at 25 °C (*p* < 0.05) (Table 5). Comparable findings have been reported by Gennari et al. [26], where immobilized *A. oryzae* β-galactosidase retained approximately 50–60% of its initial activity after 90 days of storage at 4 °C. A modest, 10% increase in storage stability was observed when the support was pretreated with glutaraldehyde or acid solution.

Overall, the immobilized lipases prepared in our study retained 77–97% of their initial activity after three months of storage at 5 °C, significantly (*p* < 0.05) outperforming the soluble enzyme forms. However, glutaraldehyde treatment generally did not improve the storage stability of the immobilized enzymes compared to the untreated complexes (Table 5). These findings suggest that adsorption of fungal lipases onto Accurel MP 1000 effectively enhances their storage stability across a wide range of temperatures, even without further chemical modification.

#### 3.2.3. Temperature Stability

**Figure 3:**
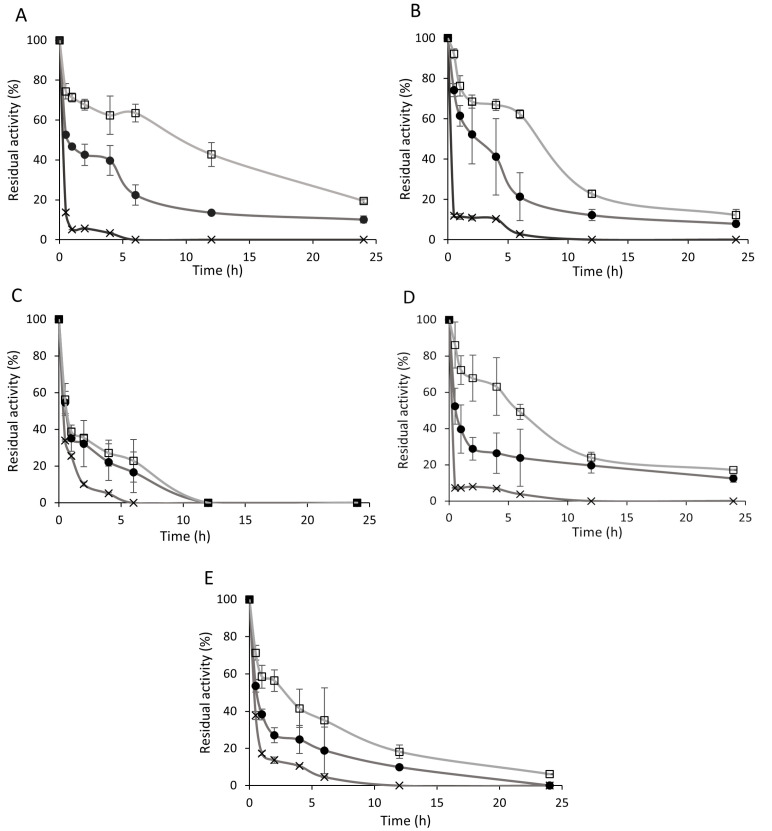
Temperature stability of soluble and immobilized *R. oryzae* (**A**), *R. niveus* (**B**), *A. niger* (**C**), *R. miehei* (**D**) and *C. rugosa* (**E**) lipases at 60 °C. ×: soluble enzyme ●: glutaraldehyde untreated immobilized enzyme; □: glutaraldehyde treated immobilized enzyme (*R. miehei* 1%, *R. niveus* and *C. rugosa* 2%, and *R. oryzae* and *A. niger* 3% (*v*/*v*) glutaraldehyde). Results are presented as averages from three replicates; error bars represent standard deviation.

The thermal stability of both the immobilized enzyme complexes and the soluble lipases was assessed by monitoring their residual activities during incubation at 60 °C for 24 h. As shown in Figure 3A–E, immobilization on the Accurel support substantially improved the thermal stability of the lipases. The soluble enzymes exhibited marked thermal sensitivity, retaining less than 30% of their initial activity within the first 30–60 min of incubation. In contrast, immobilized enzymes maintained approximately 20–40% higher residual activities under the same conditions. Furthermore, except for *A. niger* lipase, glutaraldehyde crosslinking provided an additional increase of 10–30% in residual activity compared to the non-crosslinked immobilized forms (Figure 3A,B,D,E).

The most notable enhancement in thermal stability was observed for the *Rhizopus* and *Rhizomucor* immobilized lipases. While the soluble forms of *R. oryzae*, *R. niveus*, and *R. miehei* lipases exhibited less than 5% residual activity after 6 h at 60 °C (Figure 3A,B,D), their glutaraldehyde treated immobilized counterparts retained 63.5%, 62.3%, and 49.1% of their initial activities, respectively. Following 24 h of incubation, the glutaraldehyde-crosslinked *R. oryzae* immobilized enzyme displayed the highest residual activity at 19.4% (Figure 3A). In *A. niger* lipase (Figure 3C), although the enzyme remained sensitive to elevated temperatures, immobilization on Accurel still improved its thermal stability by approximately 20%. Both soluble and immobilized enzymes demonstrated a rapid initial decline in activity, which subsequently slowed during the later stages of incubation. For most enzymes tested, no remarkable loss in activity was observed beyond 12 h of incubation.

These findings align with those of Mohammadi et al. [54], who reported enhanced thermal stability of *R. miehei* lipase immobilized on epoxy-functionalized silica particles. In their study, the soluble enzyme was fully inactivated after 4 h at 55 °C, whereas the immobilized form retained 25% of its initial activity after 6 h at 60 °C. In our experiments, the *R. miehei* immobilized enzyme exhibited 23.8% residual activity, which was further improved to 49.1% following glutaraldehyde treatment (Figure 3D).

Neither the soluble nor the immobilized enzymes demonstrated stability at 80 °C for extended periods. After 30 min of incubation at this temperature, soluble enzymes retained only 0–8.1% of their initial activities, while immobilized forms exhibited slightly higher residual activities of 7.8–21.9%. Glutaraldehyde crosslinking improved thermal resistance even at this high temperature, increasing residual activity by approximately 5–10% (19.1–27.1% residual activity). However, no detectable activity remained after 1 h of incubation at 80 °C.

The increased rigidity of enzyme structures achieved through covalent crosslinking likely enhanced resistance to denaturation under harsh conditions, such as elevated temperatures [55]. Consistently, the glutaraldehyde treated immobilized enzymes in this study demonstrated superior thermal tolerance. A similar stabilizing effect was observed for β-glucosidase enzymes immobilized on aminated agarose beads followed by glutaraldehyde crosslinking [25]. Given that many industrial processes operate at elevated temperatures, the enhancement of lipase thermostability through immobilization and crosslinking presents a valuable strategy for industrial applications [56].

#### 3.2.4. pH Stability

The stability of both soluble and immobilized enzymes was evaluated after 24 h of incubation at pH 5.5, 7.0, and 8.5 (Table 6). Immobilization onto the Accurel support improved the pH stability of *R. oryzae*, *R. miehei*, and *C. rugosa* lipases (*p* < 0.05), as reflected by the residual activities of 56.1–76.2%, compared to only 8.0–39.6% for their soluble counterparts. In contrast, soluble *R. niveus* and *A. niger* lipases exhibited inherently higher stability across the tested pH range, retaining 28.5–107.4% of their initial activity.

The immobilized *R. niveus* lipase displayed significantly greater stability at pH 5.5 (by 35–50%; *p* < 0.05) compared to the soluble enzyme. At pH 7.0 and 8.5, approximately 1.1–1.2-fold higher residual activities were observed for the immobilized *R. niveus* complex relative to the soluble form.

In contrast, immobilization of *A. niger* lipase resulted in a 23–60% reduction in residual activity compared to the soluble enzyme (Table 6). For most enzymes tested, glutaraldehyde treatment did not markedly influence pH stability compared to aldehyde free immobilization. However, in the case of the immobilized *A. niger* lipase, glutaraldehyde post-treatment significantly enhanced stability at pH 7.0 and 8.5 (*p* < 0.05), although the activity levels remained below those of the corresponding soluble enzyme (Table 6).

Several studies have highlighted the impact of immobilization on the pH stability of lipases, which often depends on both the nature of the enzyme and the immobilization strategy applied. Xie et al. [57] demonstrated that immobilization of *Thermomyces lanuginosus* lipase improved its pH stability and broadened its operational stability during reactions. Similarly, Wang et al. [58] reported that lipase from *Burkholderia ambifaria* immobilized on Eupergit C retained higher activity over a pH range of 7.0–9.0 compared to its soluble form. In another study, Monteiro et al. [59] observed that *Candida antarctica* lipase A immobilized onto halloysite nanotubes showed enhanced pH stability both acidic and alkaline pHs than its soluble counterpart enzyme. The *C. rugosa* lipase showed also improved resistance to pH between pH 7.0 and 9.0 after immobilization to chitosan beads using genipin as natural crosslinker [60]. In line with these findings, our results also confirm that adsorption onto Accurel MP 1000 generally improved the pH stability of fungal lipases tested. However, the effect of glutaraldehyde post-treatment on pH tolerance was enzyme-dependent, which aligns with the notion that both the immobilization matrix and crosslinking chemistry can have varied effects on the conformational stability of different lipases.

#### 3.2.5. Organic Solvent Stability

The stability of immobilized lipase preparations in organic solvents of varying hydrophobicity (logP ranging from −1.3 to 3.5) was assessed by incubating the complexes in concentrated solutions of DMSO, methanol, ethanol, propanol, and hexane for 4 h and 24 h. The residual activities after incubation are presented in Table 7 (4 h) and Table 8 (24 h).

The results demonstrate a clear correlation (0.693 < r < 0.988) between solvent hydrophobicity and the residual activity of the immobilized lipases. After 4 h of incubation, the lowest residual activities (2.7–17.8%) were detected in the presence of DMSO and methanol, while considerably higher residual activities (66.1–104.1%) were observed in hexane (Table 7).

Prolonged incubation for 24 h led to a complete loss of activity in DMSO and methanol, and to very low residual activities (up to 14.6% for immobilized *R. oryzae* lipase) in ethanol (Table 8). In contrast, the enzymes retained 16.0–53.2% of their initial activity in propanol and 33.9–70.4% in hexane. Glutaraldehyde crosslinking slightly improved the ethanol and hexane stability of the *A. niger* immobilized lipase, while a significant (*p* < 0.05) stabilization effect was detected for the *R. miehei* immobilized preparation in both propanol and hexane (Table 8). Conversely, glutaraldehyde treatment decreased the stability of *R. niveus* and *C. rugosa* immobilized lipases in hexane and propanol, respectively. For the other immobilized enzymes and solvents tested, glutaraldehyde treatment had no significant impact on solvent stability (Table 8).

Alnoch et al. [7] reported similar results when a lipase isolated from a metagenomic library was immobilized on Accurel MP 1000: the enzyme showed very low residual activity in methanol and DMSO. However, they did not observe a consistent correlation between solvent hydrophobicity and enzyme stability, noting that high stability (over 80%) was retained even in polar solvents such as ethanol and acetone. Generally, polar solvents are known to destabilize lipases by disrupting the essential hydration layer on the enzyme surface [54].

## 4. Conclusions

This study evaluated the immobilization of fungal lipases from *R. oryzae*, *R. niveus*, *A. niger*, *R. miehei* and *C. rugosa* on the hydrophobic support Accurel MP 1000 under varying conditions. The main findings are: (i) *R. miehei* and *C. rugosa* lipases resulted in the highest immobilized activity among the tested enzymes, (ii) immobilization yields reached 47–83% at high enzyme concentrations (1 mg/mL), but activity yields were reduced, likely due to support saturation or diffusion limitations, (iii) at lower enzyme powder concentrations (0.01–0.001 mg/mL), immobilization efficiency exceeded 80%, with strong dependence on acidic pH (4.5 or 5.5) and low temperature (5 °C), (iv) glutaraldehyde treatment (at glutaraldehyde concentration up to 3%) reduced initial activities by only about 10–40%, (v) biocatalysts operated without loss of initial activity during consecutive 7–10 reaction cycles under reaction conditions studied, (vi) storage stability was improved, with activity preserved for up to three months, (vii) thermal stability increased, with tolerance up to 60 °C, and pH stability was maintained at pH 5.5, 7.0 and 8.5, with residual activities of 56.1–76.2% after 24 h, (viii) a clear correlation was observed between solvent hydrophobicity and residual activity, (ix) glutaraldehyde treatment modulated solvent tolerance differently depending on the solvent and enzyme type. In conclusion, while immobilization of the applied fungal lipases on Accurel MP 1000, with or without glutaraldehyde treatment, provided significant gains in stability and reusability, further research is necessary to evaluate their catalytic activity in reactions operated at industrial scale.

## Figures and Tables

**Table 1:** Detailed data of immobilization of commercial fungal lipases adsorbed in different concentrations to Accurel MP 1000 support. The amount of support was constant at 200 mg.

Lipase Source	Lipase Solution Concentration (mg/mL) ^1^	Enzyme Activity (U/mL)	Protein Concentration (µg/mL)	Overall Time of 100% Immobilization Efficiency (min)	Immobilized Activity (U/mg Support)	Activity Yield (%)
*R. oryzae*	1	1696.73	21.8	90	2.03 (68) a	2.39
	0.1	169.67	2.18	30	0.47 (4) ab	5.5
	0.01	16.97	0.21	<15	0.21 (5) b	24.7
	0.001	1.7	0.02	<15	0.10 (10) b	121.2
*R. niveus*	1	212.17	143	360	0.41 (29) a	3.87
	0.1	21.22	14	45	0.23 (22) ab	21.7
	0.01	2.12	1.4	<15	0.12 (8) b	115.2
*A. niger*	1	238.5	78.2	360	4.16 (30) a	34.88
	0.1	23.9	7.8	60	0.51 (29) b	42.77
	0.01	2.39	0.78	<30	0.06 (17) b	50.21
*R. miehei*	1	2231	82.7	1440 ^2^	25.07 (13) a	22.47
	0.1	223.1	8.27	240	3.17 (35) b	28.42
	0.01	22.31	0.83	60	0.37 (19) b	33.18
	0.001	2.23	0.083	<15	0.08 (38) b	71.75
*C. rugosa*	1	2703	91.3	1440 ^2^	21.18 (2) a	15.67
	0.1	270.3	9.13	120	2.84 (12) b	21.01
	0.01	27.03	0.91	90	0.54 (4) c	37.78
	0.001	2.7	0.091	<15	0.11 (36) c	81.48

^1^ Enzyme powders were dissolved in 25 mM pH 7.0 phosphate buffer (*R. oryzae*, *R. niveus*, *R. miehei* and *C. rugosa* lipases) or 25 mM pH 4.5 acetate buffer (*A. niger* lipase). ^2^ Immobilization efficiency of the 1 mg/mL enzyme solutions was 93% for the *R. miehei* lipase and 97% for the *C. rugosa* lipase. Average values from three tests (% coefficient of variation). Values for each enzyme with different letters are significantly different according to one-way ANOVA followed by Tukey’s multiple comparison test (*p* < 0.05).

**Table 2:** Relative protein content of the supernatant during immobilization of 1 mg/mL lipase solutions.

Lipases	Relative Protein Content (%)						
	Incubation Time (h)						
	0	0.5	1	2	4	6	24
*R. oryzae*	100	76.37	35.2	21.43	n. d. ^1^	n. d.	n. d.
*R. niveus*	100	54.25	41.14	31.79	27.9	25.21	16.06
*A. niger*	100	66.93	59.85	51.75	48.34	39.32	34.23
*R. miehei*	100	78.61	74.71	69.11	56.8	n. d.	53.21
*C. rugosa*	100	59.48	42.29	39.27	32.08	n. d.	27.99

^1^ No data.

**Table 3:** Effect of reaction temperature and pH on lipase immobilization to Accurel MP 1000.

Lipase Source	Circumstances of the Immobilization (Temperature, pH) ^1^	Time of 100% Immobilization Efficiency (min)	Relative Immobilized Activity (%) ^2^
*R. oryzae*	Standard	<30	100 (0) ab
	25 °C, pH 5.5	<60	88.3 (2) a
	25 °C, pH 8.5	<30	62.7 (2) c
	5 °C, pH 7.0	<60	109.9 (11) b
*R. niveus*	Standard	<45	100 (0) a
	25 °C, pH 5.5	<90	77.8 (2) b
	25 °C, pH 8.5	<45	45.4 (19) c
	5 °C, pH 7.0	<60	82.2 (12) b
*A. niger*	Standard (pH 4.5)	<60	100 (0) a
	25 °C, pH 7.0	<120	12.1 (26) b
	5 °C, pH 4.5	<90	80.0 (6) c
*R. miehei*	Standard	<60	100 (0) ab
	25 °C, pH 5.5	<90	144.0 (5) ac
	25 °C, pH 8.5	<90	62.5 (12) b
	5 °C, pH 7.0	<90	181.8 (21) c
*C. rugosa*	Standard	<90	100 (0) a
	25 °C, pH 5.5	<120	78.2 (6) b
	25 °C, pH 8.5	<90	22.7 (26) c
	5 °C, pH 7.0	<120	109.0 (5) a

^1^ Standard immobilization conditions for *R. oryzae* and *R. niveus* lipases: 0.1 mg/mL enzyme, 25 °C, pH 7.0; for *A. niger* lipase: 0.1 mg/mL enzyme, 25 °C, pH 4.5; for *R. miehei* and *C. rugosa* lipases: 0.01 mg/mL enzyme, 25 °C, pH 7.0. Buffers: 50 mM sodium phosphate (pH 7.0), 50 mM sodium acetate (pH 4.5 and 5.5), 50 mM Tris-HCl (pH 8.5). ^2^ Average values from three tests (% coefficient of variation). Values for each enzyme with different letters are significantly different according to one-way ANOVA followed by Tukey’s multiple comparison test (*p* < 0.05).

**Table 4:** Effect of glutaraldehyde treatment on the activity of immobilized fungal lipases. Activity of initial enzyme–Accurel MP 1000 complexes without any treatment incubation was taken as 100% (Initial biocatalyst). Activity of enzyme–Accurel MP 1000 complexes incubated under glutaraldehyde-free treatment conditions was taken as the control.

	Relative Activity (%) of Immobilized Fungal Lipases ^3^				
	*R. oryzae*	*R. niveus*	*A. niger*	*R. miehei*	*C. rugosa*
Initial biocatalyst	100 (0) a	100 (0) a	100 (0) a	100 (0) a	100 (0) a
Control ^1^	76.0 (14) a	84.3 (11) a	64.8 (17) b	75.3 (5) b	88.0 (4) ab
Treated with glutaraldehyde at various concentrations (*v*/*v*%) ^2^					
1%	76.8 (8) a	90.7 (1) a	52.4 (14) b	83.6 (13) ab	75.1 (17) b
2%	86.3 (18) a	95.6 (12) a	60.9 (6) b	78.4 (4) b	80.2 (13) ab
3%	93.7 (17) a	93.3 (2) a	71.6 (19) b	73.5 (13) b	76.9 (6) b

Standard immobilization conditions for *R. oryzae* and *R. niveus* lipases: 0.1 mg/mL enzyme, 25 °C, pH 7.0; for *A. niger* lipase: 0.1 mg/mL enzyme, 25 °C, pH 4.5; for *R. miehei* and *C. rugosa* lipases: 0.01 mg/mL enzyme, 25 °C, pH 7.0. ^1^ After standard immobilization, samples were incubated in 50 mM sodium phosphate buffer (pH 7.0) at 25 °C for 1 h, under mild stirring. ^2^ After standard immobilization, samples were incubated in a solution of 1–3% (*v*/*v*) glutaraldehyde in 50 mM sodium phosphate buffer (pH 7.0) at 25 °C for 1 h, under mild stirring. ^3^ Average values from three tests (% coefficient of variation). Values for each enzyme with different letters are significantly different according to one-way ANOVA followed by Tukey’s multiple comparison test (*p* < 0.05).

**Table 5:** Comparative evaluation of the stability of soluble and immobilized fungal lipases after 90 days of storage at −20 °C, 5 °C and 25 °C temperatures.

Temperature	Residual Activity of Fungal Lipases (%)														
	*R. oryzae*			*R. niveus*			*A. niger*			*R. miehei*			*C. rugosa*		
	Soluble ^1^	Im ^2^	ImG ^3^	Soluble	Im	ImG	Soluble	Im	ImG	Soluble	Im	ImG	Soluble	Im	ImG
−20 °C	5.3 (30) a	93.3 (6) b	98.0 (16) b	14.2 (16) a	88.1 (2) b	75.7 (5) c	3.5 (31) a	100.9 (4) b	91.2 (5) c	22.1 (1) a	85.8 (5) b	84.1 (14) b	8.12 (14) a	94.4 (5) b	96.8 (15) b
5 °C	n. d. ^4^ a	77.7 (10) b	89.3 (13) b	n. d. a	81.3 (10) b	79.3 (11) b	n. d. a	97.3 (5) b	86.2 (1) c	n. d. a	96.3 (13) b	86.2 (19) b	n. d. a	86.2 (10) b	80.4 (14) b
25 °C	n. d. a	66.6 (4) b	84.1 (4) c	n. d. a	80.1 (10) b	83.7 (14) b	n. d. a	73.1 (11) b	54.1 (13) c	n. d. a	86.5 (13) b	78.4 (12) b	n. d. a	67.1 (6) b	84.8 (14) b

^1^ Soluble lipase in 50 mM phosphate buffer (pH 7.0) solution. ^2^ Glutaraldehyde untreated immobilized lipases. ^3^ Glutaraldehyde treated immobilized lipases. ^4^ Not detected. Average values from three tests (% coefficient of variation). Values for each enzyme and corresponding temperatures with different letters are significantly different according to one-way ANOVA followed by Tukey’s multiple comparison test (*p* < 0.05).

**Table 6:** Stability of soluble and immobilized fungal lipases in 50 mM acetate (pH 5.5), phosphate (pH 7.0) or Tris-HCl (pH 8.5) buffers during 24 h incubation at 5 °C.

pH	Residual Activity of Fungal Lipases (%)														
	*R. oryzae*			*R. niveus*			*A. niger*			*R. miehei*			*C. rugosa*		
	Soluble ^1^	Im ^2^	ImG ^3^	Soluble	Im	ImG	Soluble	Im	ImG	Soluble	Im	ImG	Soluble	Im	ImG
pH 5.5	19.6 (30) a	68.0 (24) b	62.4 (17) b	28.5 (54) a	79.1 (5) b	66.8 (14) b	107.4 (18) a	40.5 (26) b	77.3 (18) ab	31.4 (25) a	69.7 (5) b	64.6 (7) b	39.6 (19) a	65.0 (2) b	67.4 (15) b
pH 7.0	12.6 (64) a	62.5 (14) b	72.9 (15) b	59.1 (30) a	67.3 (20) a	71.8 (7) a	103.4 (3) a	41.8 (18) b	77.5 ± (23) a	25.9 (27) a	65.8 (15) b	71.8 (5) b	31.5 (21) a	69.8 (4)	56.1 (3) c
pH 8.5	8.0 (44) a	63.4 (17) b	76.2 (14) b	51.2 (21) a	71.9 (22) a	69.6 (18) a	105.9 (4) a	42.9 (19) b	74.5 (22) c	36.3 (25) a	69.1 (1) b	65.1 (7) b	36.7 (34) a	74.2 (4) b	56.9 (11) ab

^1^ Soluble lipase. ^2^ Glutaraldehyde untreated immobilized lipases. ^3^ Glutaraldehyde treated immobilized lipases. Average values from three tests (% coefficient of variation). Values for each enzyme and corresponding pH with different letters are significantly different according to one-way ANOVA followed by Tukey’s multiple comparison test (*p* < 0.05).

**Table 7:** Organic solvent stability of the immobilized lipases incubated in solvents representing a logP value range from −1.3 to 3.5 at 5 °C during 4 h.

Organic solvents (logP)	Residual Activity of Fungal Lipases (%)									
	*R. oryzae*		*R. niveus*		*A. niger*		*R. miehei*		*C. rugosa*	
	Im ^1^	ImG ^2^	Im	ImG	Im	ImG	Im	ImG	Im	ImG
DMSO (−1.3)	15.9 (11)	14.4 (8)	9.5 (8)	5.3 (4) **	12.0 (11)	6.4 (14) **	16.9 (9)	16.8 (11)	14.7 (16)	17.8 (11)
Methanol (−0.76)	10.6 (8)	10.3 (10)	4.4 (9)	8.7 ± (13) **	9.5 (8)	2.7 (22) **	11.8 (6)	13.2 (10)	6.4 (14)	6.3 (11)
Ethanol (−0.3)	28.3 (23)	33.3 (15)	25.9 (5)	18.8 (3) **	10.9 (6)	19.7 (11) **	16.3 (16)	20.1 (43)	2.7 (4)	4.8 (35)
*n*-Propanol (0.25)	50.2 (3)	56.7 (1) **	45.3 (15)	53.4 (0)	36.7 (5)	38.8 (14)	37.9 (12)	55.6 (1) **	45.5 (19)	33.0 (3) *
*n*-Hexane (3.5)	76.0 (22)	87.1 (11)	94.1 (25)	79.1 (28)	84.3 (9)	104.1 (22)	66.1 (12)	80.6 (20)	90.6 (21)	88.7 (19)

^1^ Glutaraldehyde untreated immobilized lipases. ^2^ Glutaraldehyde treated immobilized lipases. Average values from three tests (% coefficient of variation). Asterisks indicate significant differences between the glutaraldehyde treated and untreated immobilized enzymes according to a multiple *t*-test performed by GraphPad Prism version 8.00, FDR (Q = 10%), * *p* < 0.05, ** *p* < 0.01.

**Table 8:** Organic solvent stability of the immobilized lipases incubated in solvents representing a logP value range from −1.3 to 3.5 at 5 °C during 24 h.

Organic Solvents (logP)	Residual Activity of Fungal Lipases (%)									
	*R. oryzae*		*R. niveus*		*A. niger*		*R. miehei*		*C. rugosa*	
	Im ^1^	ImG ^2^	Im	ImG	Im	ImG	Im	ImG	Im	ImG
DMSO (−1.3)	n. d. ^3^	n. d.	n. d.	n. d.	n. d.	n. d.	n. d.	n. d.	n. d.	n. d.
Methanol (−0.76)	n. d.	n. d.	n. d.	n. d.	n. d.	n. d.	n. d.	n. d.	n. d.	n. d.
Ethanol (−0.3)	14.6 (62)	8.9 (46)	1.5 (33)	2.5 (52)	4.7 (36)	8.4 (29)	6.8 (32)	4.4 (36)	n. d.	n. d.
*n*-Propanol (0.25)	41.0 (2)	42.9 (12)	35.1 (25)	41.4 (11)	34.1 (29)	28.0 (6)	28.7 (7)	53.2 (13) **	33.6 (9)	16.0 (16) **
*n*-Hexane (3.5)	50.9 (2)	50.8 (12)	57.2 (9)	33.9 (9) **	52.8 (12)	65.4 (11)	45.4 (9)	70.4 (11) **	47.8 (2)	51.6 (17)

^1^ Glutaraldehyde untreated immobilized lipases. ^2^ Glutaraldehyde treated immobilized lipases. ^3^ Not detected. Average values from three tests (% coefficient of variation). Asterisks indicate significant differences between the glutaraldehyde treated and untreated immobilized enzymes according to a multiple *t*-test performed by GraphPad Prism version 8.00, FDR (Q = 10%), ** *p* < 0.01.

## Data Availability

The original contributions presented in the study are included in the article, further inquiries can be directed to the corresponding author.
